# Optimization of L‐Arginine to Meet the Dietary Requirement of Juvenile Coho Salmon (*Oncorhynchus kisutch*)

**DOI:** 10.1155/anu/6614910

**Published:** 2026-04-17

**Authors:** Leyong Yu, Abdur Rahman, Hairui Yu, Govindhrajan Sattanathan, Lingyao Li, Maida Mushtaq, Muhammad Younus, Muhammad Khan, Muhammad Uzair Akhtar

**Affiliations:** ^1^ College of Fisheries, Huazhong Agricultural University, Wuhan, China, hzau.edu.cn; ^2^ Key Laboratory of Biochemistry and Molecular Biology in Universities of Shandong (Weifang University), Weifang Key Laboratory of Coho Salmon Culturing Facility Engineering, Institute of Modern Facility Fisheries, College of Biology and Oceanography, Weifang University, Weifang, China, wfu.edu.cn; ^3^ Weifang Key Laboratory of Salmon and Trout Health Culture, Conqueren Leading Fresh Science and Technology Inc., Ltd., Weifang, China; ^4^ Department of Animal Sciences, University of Veterinary and Animal Sciences, Lahore, Pakistan, uvas.edu.pk; ^5^ Shandong Collaborative Innovation Center of Coho Salmon Health Culture Engineering Technology, Shandong Conqueren Marine Technology Co., Ltd., Weifang, China; ^6^ Yunnan Animal Science and Veterinary Institute, Kunming, China; ^7^ Department of Pathology, University of Veterinary and Animal Sciences, Lahore, Pakistan, uvas.edu.pk; ^8^ Department of Animal Nutrition, Cholistan University of Veterinary and Animal Sciences, Bahawalpur, Pakistan, cuvas.edu.pk

**Keywords:** amino acids, coho salmon, growth performance, L-arginine, protein deposition, protein efficiency ratio

## Abstract

The current study aimed to determine the dietary L‐arginine requirements of Coho salmon by evaluating key performance indicators, including growth rate, feed efficiency, nitrogen retention, and body composition. A total of 1800 juvenile coho salmon (*Oncorhynchus kisutch*) (initial weight: 0.32 ± 0.01 g) were randomly assigned to six isonitrogenous and isoenergetic dietary treatments containing different L‐arginine levels, with three replicates per group (100 fish per 240 L tank) by following a completely randomized design. The L‐arginine supplementation levels were 0% (D‐1), 0.50% (D‐2), 1% (D‐3), 1.50% (D‐4), 2% (D‐5), and 2.50% (D‐6) of the feed, resulting in cumulative dietary arginine concentrations of 2.18%, 2.60%, 2.97%, 3.41%, 3.79%, and 4.16% in the feed, respectively. Following a 14‐day acclimatization period, the feeding trial was conducted for 84 days. The survival rates were similar across the groups (*p* > 0.05). However, growth performance parameters, including body weight gain and specific growth rates (SGRs), were increased with dietary arginine levels (*p* < 0.05). Arginine supplementation of 1% was the best compared with other levels in terms of feed conversion ratio (FCR) (*p* < 0.05). The group fed on the D‐3 level had the highest protein efficiency ratio (1.61 ± 0.03), which differed significantly from D‐1 (*p* < 0.05). Body protein deposition (BPD) was also highest in D‐3 (23.93 ± 0.50%), with significant differences between lower and higher dietary L‐arginine levels (*p* < 0.05). Fish in the D‐1 and D‐2 groups exhibited significantly lower whole‐body arginine levels than other treatments. Lysine content was considerably higher in the D‐3 and D‐4 fed groups, and valine content was significantly higher in the D‐3 group than D‐1 and D‐2 groups (*p* < 0.05), while other essential amino acid levels remained unaffected (*p* > 0.05). The quadratic regression analysis revealed that the estimated cumulative total dietary L‐arginine requirement for optimal FCR, SGR, BPD, and protein efficiency ratio of coho salmon was 3.29%, 3.19%, 3.33%, and 3.26%, respectively. Based on the findings, dietary supplementation with L‐arginine at an optimal level of ~1% significantly enhances growth performance, feed conversion efficiency, protein utilization, and the deposition of essential amino acids, particularly arginine, lysine, and valine, in juvenile salmon. However, supplementation beyond this level yields diminishing benefits, likely due to metabolic saturation or feedback regulation mechanisms.

## 1. Introduction

The growth and health of farmed fish depend on a well‐balanced diet that provides essential nutrients, particularly amino acids, which serve as the building blocks of proteins and play critical roles in metabolism [1]. L‐arginine, an essential amino acid in fish [2, 3], is involved in several vital physiological functions, including protein synthesis, immune modulation, nitrogen excretion, and energy metabolism [2, 4, 5]. Unlike some vertebrates that can endogenously synthesize sufficient amounts of L‐arginine via the urea cycle, fish generally have a limited ability to produce this amino acid, making dietary supplementation necessary with high plant‐protein based diets, for optimal growth and metabolic function [6]. Therefore, arginine is known as an indispensable amino acid, and its dietary supplementation is essential to enhance the performance of the fish.

Understanding the specific L‐arginine requirements of commercially valuable fish species is crucial for formulating nutritionally efficient and cost‐effective aqua feeds. Previously, a few researches have been conducted to optimize the arginine content in fish species and reported the optimum arginine levels of 2.03% in terms of specific growth rate (SGR) for blunt snout bream [7] and 2.54% in terms of growth performance for juvenile red sea bream [8]. Nile tilapia fed a standard arginine level also exhibited improved growth performance and feed efficiency following dietary supplementation with N‐carbamylglutamate [9]. Nevertheless, arginine requirements of fish varies with species and environmental conditions in terms of differences in metabolic pathways, growth rates, and nitrogen utilization efficiency [10]. For instance, previous studies on arginine requirements were conducted mostly with 1.3%–5.6% of CP in yellow perch diet and 2.6%–9.9% of CP in grass carp diet, while arginine requirements of 7.1% for black sea bream, 3.5% for trout, 5.1% for Nile tilapia, 3.4% for yellow perch, and 3.1% for Indian major carp were reported [10]. Similarly, the arginine requirements of 2.8% for yellow groupers [11], 2.17% for grass carp [12], 2.85% for juvenile cobia [13], 2.38% for juvenile yellow catfish [14], 1.84% for blunt snout bream [15], 1.5%–2.2% for Atlantic salmon [16], 2.2%–2.4% for chinook salmon [17], 1.4%–1.9% for rainbow trout [18], 1.8%–2.5% for coho salmon [17], and 1.80% for Jian carp [19] as percent of dry matter has been previously. Ahmed et al. [20] reported that growth performance exhibited a positively improving linear trend with increasing dietary arginine supplies as fish fed diet containing arginine up to 1.75% showed the highest body protein content, lowest moisture, and moderate fat levels. However, arginine requirements of coho salmon (*Oncorhynchus kisutch*) have not yet been explored.

Coho salmon is a species of high economic importance in global aquaculture [21], valued for its rapid growth, high‐quality flesh, and adaptability to different rearing environments [22]. Like other salmonids, coho salmon require a well‐balanced amino acid profile to support their growth and physiological functions [23]. L‐arginine, in particular, plays a unique role as a precursor for the synthesis of nitric oxide, polyamines, creatine, and other metabolites essential for cardiovascular function, stress response, and immune defense [24, 25]. Inadequate dietary L‐arginine levels can result in reduced growth performance, impaired feed conversion efficiency, weakened immune responses, and disruptions in nitrogen metabolism, ultimately affecting overall health and productivity [3].

However, limited data are available on the specific L‐arginine requirements of coho salmon, particularly during the juvenile stage, highlighting the need for targeted research. Given the increasing demand for sustainable and nutritionally optimized aqua feeds, defining the precise dietary L‐arginine needs of coho salmon is essential for improving feed formulations and reducing nitrogen waste in aquaculture systems. The present study aimed to determine the dietary L‐arginine requirements of coho salmon by evaluating key performance indicators, including growth rate, feed efficiency, nitrogen retention, and body composition across different dietary L‐arginine levels. This research will help optimize the L‐arginine requirements of coho salmon and support the development of more efficient and sustainable feeding strategies in salmonid aquaculture.

## 2. Materials and Methods

The ethical approval for the current experiment was granted by the Institutional Animal Care and Use Committee, Institute of Modern Facility Fisheries of Weifang University, China (No. 20220516004).

### 2.1. Experimental Design, Site, and Diet Preparation

The experiment was conducted at the Coho Salmon Fry Breeding Center, Shandong Province Coho Salmon Engineering Technology Collaborative Innovation Center (Linyi, China). Induced bred coho salmon juveniles were obtained from the center in April of 2023, and fed a commercial microdiet (contained 55% crude protein and 16% crude lipid) for salmon fry purchased from Shandong Conqueren Marine Technology Co., Ltd. (Weifang, China). After a 2‐week adaptation period, total 1800 healthy juveniles (initial weight: 0.321 ± 0.005 g) were randomized into 18 groups (100 fish per group) and assigned to six dietary treatments containing L‐arginine levels (*n* = 3 groups/level) by following a completely randomized design. The L‐arginine supplementation levels were 0.00% (D‐1), 0.50% (D‐2), 1.00% (D‐3), 1.50% (D‐4), 2.00% (D‐5), and 2.50% (D‐6). Each group was housed in a 240‐L rectangular plastic tank (80 cm × 60 cm × 60 cm) within an indoor nursery system (700 cm × 500 cm × 150 cm). Tanks were equipped with bottom drainage, and water was supplied from an underground spring, pre‐treated through sedimentation, sand filtration, and final filtration using a filter bag.

Six experimental diets (D‐1 to D‐6) were formulated to contain increasing levels of L‐arginine, with three replicates per treatment. The ingredient and chemical composition of the experimental diets are presented in Table 1. Essential amino acid profiles of the ingredients used are presented in Table 2. To match the essential amino acid profile of coho salmon eggs, L‐crystalline amino acids (excluding L‐arginine) were added to the diets, ensuring that all six diets were iso‐energetic with similar protein contents. The L‐arginine content in Diet 1 was 2.18% of feed dry matter (3.93% of feed protein). Diets 2–6 were adjusted for the supplemental levels at 0.4% increments in cumulative dietary arginine supplies, resulting in cumulative dietary L‐arginine levels of 2.60%, 2.97%, 3.41%, 3.79%, and 4.16% of feed dry matter, respectively. To maintain nitrogen balance, additional crystalline amino acids (excluding arginine) were added to keep the essential amino acid composition consistent. Glycine adjustments ensured that all diets remained isonitrogenous and isoenergetic. The pH of the feeds was adjusted to 7.0–7.5 using 6 N NaOH. The feed ingredients were ground into fine powder through a 48 µm mesh, and the methodology used to prepare the microdiet is patent pending. The diets were processed into pellets (150–425 µm) by following the established protocol of [26] packaged in aluminum foil pouches, and stored at −20°C until use.

**Table 1:** Experimental diets formulation and their chemical composition on a dry basis.

Item	Treatments^a^					
	D‐1	D‐2	D‐3	D‐4	D‐5	D‐6
Ingredient (%)						
Fish meal	10.00	10.00	10.00	10.00	10.00	10.00
Shrimp meal	20.00	20.00	20.00	20.00	20.00	20.00
Chicken by‐product meal	10.00	10.00	10.00	10.00	10.00	10.00
Corn gluten meal	10.00	10.00	10.00	10.00	10.00	10.00
Wheat gluten meal	10.00	10.00	10.00	10.00	10.00	10.00
Beer yeast	5.00	5.00	5.00	5.00	5.00	5.00
L‐Arginine	0.00	0.50	1.00	1.50	2.00	2.50
Glycine	3.50	2.80	2.10	1.40	0.70	0.00
Cellulose	0.00	0.20	0.40	0.60	0.80	1.00
Amino acid mixture	9.34	9.34	9.34	9.34	9.34	9.34
Alpha‐starch	8.73	8.73	8.73	8.73	8.73	8.73
Soybean oil	5.00	5.00	5.00	5.00	5.00	5.00
Fish oil	5.00	5.00	5.00	5.00	5.00	5.00
Soy lecithin	1.00	1.00	1.00	1.00	1.00	1.00
Dicalcium phosphate	1.00	1.00	1.00	1.00	1.00	1.00
Vitamins premix^b^	0.50	0.50	0.50	0.50	0.50	0.50
Choline chloride	0.30	0.30	0.30	0.30	0.30	0.30
Mineral premix^c^	0.50	0.50	0.50	0.50	0.50	0.50
Vitamin C	0.10	0.10	0.10	0.10	0.10	0.10
Antioxidant^d^	0.03	0.03	0.03	0.03	0.03	0.03
Chemical composition (3 samples/treatment)						
Crude protein	54.97	54.95	54.84	55.11	55.13	55.07
Ether extract (%)	15.86	16.28	16.16	15.96	16.10	16.02
Ash (%)	9.52	9.37	9.65	9.44	9.78	9.33
Arginine (% of diet)	2.18	2.60	2.97	3.41	3.79	4.16
Arginine (% dietary protein)	3.93	4.73	5.47	6.17	6.89	7.63
Gross energy (kJ g^−1^ DM)	21.70	21.89	22.08	22.18	21.96	22.04

^a^Treatments = Dietary L, arginine levels of 2.18% (D‐1), 2.60% (D‐2), 2.97% (D‐3), 3.41% (D‐4), 3.79% (D‐5), and 4.16% (D‐6).

^b^Composition as IU or g/kg of the premix: cholecalciferol 4000 IU, retinal palmitate 10,000 IU, menadione 22 g, D, calcium pantothenate 150 g, cyanocobalamin 0.3 g, thiamine HCl 40 g, DL, alpha‐tocopherol acetate 75 g, riboflavin 30 g, pyridoxine‐HCl 20 g, DL, alpha‐tocopherol 4000 IU‐HCl 20 g, D, biotin 1 g, meso‐inositol 300 g, folic acid 15 g, and niacin 200 g.

^c^Composition as g/kg of premix: AIK(SO_4_)_(2)_‐12H_2_O, 124.0; CaCl_2_, 17,880.0; CoCl_(2)_‐6H_2_O, 49.0; FeSO_(4_)‐7H_2_O, 707.0; KCl, 1192.0 KI, 5.0; MgSO_(4_)‐7H_2_O, 4317.0; MnSO_(4_)‐4H_2_O, 31.0; NaCl, 4934.0; Na_2_SeO_(3_)‐H_2_O, 3.0; ZnSO_(4_)‐7H_2_O, 177.0; Ca(H_2_PO_4_)_(2_)‐H_2_O, 12,457.0; KH_2_PO_4_, 9930.0.

^d^Dihydropyridine was purchased from Shanghai Yuanye Biotechnology Co., Ltd. (Shanghai, China).

**Table 2:** Essential amino acid composition on a dry basis (%) of ingredients used for experimental diets’ formulation.

Ingredient	Lysine	Histidine	Methionine	Phenylalanine	Leucine	Isoleucine	Threonine	Valine	Arginine
Fish meal	0.6	0.18	0.23	0.36	0.51	0.33	0.35	0.35	0.47
Shrimp meal	0.88	0.23	0.32	0.54	0.91	0.6	0.56	0.64	0.66
Chicken by product	0.28	0.1	0.09	0.19	0.32	0.16	0.2	0.21	0.38
Corn gluten meal	0.12	0.13	0.15	0.46	1.03	0.43	0.3	0.33	0.22
Wheat gluten meal	0.16	0.19	0.14	0.35	0.33	0.6	0.23	0.37	0.33
Beer yeast	0.18	0.06	0.04	0.22	0.25	0.15	0.12	0.18	0.12
Amino acid mixture	1.84	0.7	1.31	0.66	1.08	0.8	0.94	1.74	0.00

*Note:* Tryptophan was not analyzed.

### 2.2. Fish Husbandry

Water quality parameters were maintained at 16.0 ± 1.0°C, pH 7.3 ± 0.2, and dissolved oxygen above 7.0 mg O_2_/L. The fish were exposed to natural light throughout the trial. Five fish were collected for initial body composition before the trial period, while the experimental fish were fed their respective diets daily four times (0700, 1030, 1400, 1730 h) to satiety for 84 days. Additionally, water was changed in each tank on daily basis. During the acclimatization period, health status was monitored regularly including the visual coloration assessment of skin and gills, detection of any external wounds, and any signs of illness. Fish exhibiting completely normal skin, gills, and overall health with no visible injury or infection, and normal swimming behavior in flowing water were enrolled in the current experiment.

### 2.3. Growth Performance Measurements

Before starting the feeding trial, initial body weights were calculated based on a tank biomass weight divided by the number of fish in the tank. The survival rate was estimated at the end of the experiment and fish’s body weight was measured before morning feeding. Growth performance parameters, including feed conversion ratio (FCR), hepatosomatic index (HSI), feed intake, SGR, body protein deposition (BPD), Fulton’s condition factor (K) [27], and viscerosomatic index (VSI) were then calculated using the formulas previously described by Yu et al. [28] and Yu et al. [23].

SGR %/day=100×Infinal weight− Ininitial weight/days of growth trial,

BPD %=100×BWfg× CPf %− BWig× CPi %/FIg× FP%,

Protein efficiency ratio PER,%=BWf g− BWi g/FIg× FP%,

Survival rate %=100×number of fish survived/initial number of fish,

K % g/cm3=100×weightg/total length in cm3,

HSI %=100×final liver weightg/final body weightg,

VSI %=100×final visceral weightg/final body weightg,

where BW_f_ and BW_i_ represent the final and initial body weights, CP_f_ and CP_i_ denote the final and initial carcass protein contents of the fish, respectively; FI refers to feed intake, and FP indicates the protein content of the feed.

### 2.4. Sample Collection and Laboratory Analysis

After withholding from feed for 24 h, five fish from each replicate (*n* = 15/treatment) were placed in bucket and anesthetized using tricane methanesulphate (30 mg/L of MS‐222; Wuhan Biocar Pharmaceutical Co., Ltd., Wuhan, China) and immediately frozen to euthanize with liquid nitrogen at −80°C at tank site and then transferred to laboratory to store at −20°C in refrigerator for subsequent analysis [29]. After taking the measurements of body length and weight, fish were homogenized using a meat mincer for chemical analysis of the whole‐body composition. Feed samples were collected weekly, whereas feed and meat samples were analyzed for proximate composition as reported in our previous study [23, 30]. Briefly, moisture, crude protein, crude fat, and ash contents were analyzed according to the methods of AOAC [31]. Total energy content was measured using a Parr 1281 automated oxygen bomb calorimeter (Parr, Moline, IL, USA). The L‐arginine and other amino acid concentrations in feed and fish samples were determined using A300 automatic amino acid analyzer (PMA GmbH, Germany). Briefly, after mincing, lyophilization, and grinding, 5 mL performic acid was used to oxidize a 100 mg sample for 16 h. Subsequently, 15 mL of 6 N phenolic HCl was used for the hydrolysis and hydrolysate was then filtered after centrifugation to estimate the amino acid profile of the samples [30].

### 2.5. Statistical Analysis

Raw data was subjected to QQ plots using SPSS 11.5 (SPSS Inc., Chicago, IL, USA) for normality, and data was log transformed before analysis. One‐way ANOVA was run using the SPSS software to assess differences among treatments. Tukey’s multiple comparison test was applied when significant differences were detected (*p* < 0.05). Quadratic regression analysis was used to predict the optimal cumulative total requirement of arginine for best SGR, FCR, PER, and BPD achievement. The dosage optimization was carried out with the following regression model (GraphPad Prism 9.1.0).

Y=L+U×R−X×I,

where, *Y* is dependent variable, *L* is theoretical maximum, *U* is rate constant, *R* is the requirement, *X* is independent variable, *I* is 1 if *X* < *R* or *I* is 0 if *X* > *R*.

## 3. Results

### 3.1. Growth Performance

**Figure 1:**
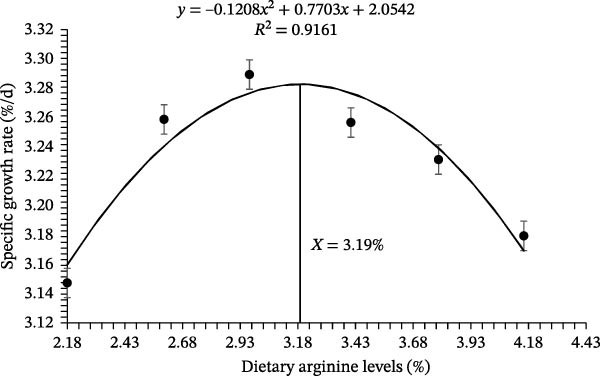
Quadratic regression analysis of the mean specific growth rate estimated that the optimum cumulative total dietary arginine requirement is 3.19% for the coho salmon juveniles.

**Figure 2:**
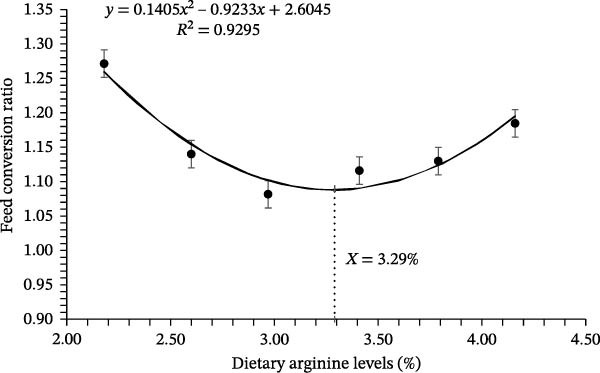
Quadratic regression analysis of the mean feed conversion ratio estimated that the optimum cumulative total dietary arginine requirement is 3.29% for the coho salmon juveniles.

**Figure 3:**
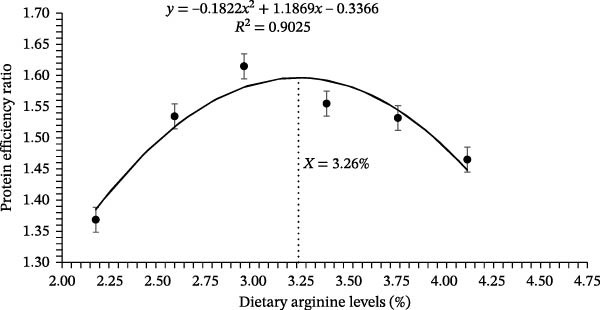
Quadratic regression analysis of the mean protein efficiency ratio estimated that the optimum cumulative total dietary arginine requirement is 3.26% for the coho salmon juveniles.

**Figure 4:**
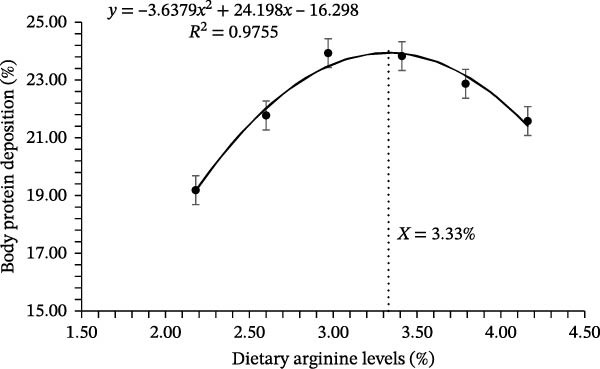
Quadratic regression analysis of the body protein deposition rate estimated that the optimum cumulative total dietary arginine requirement is 3.33% for the coho salmon juveniles.

Growth performance‐related results of coho salmon juveniles are presented in Table 3. The survival rate of juveniles ranged from 94.33% to 97.67%, with no significant differences among dietary arginine supplemented groups (*p* > 0.05). Initial body weight was consistent across all groups (*p* > 0.05), with values between 0.318 and 0.323 g. However, the final BW was significantly affected by dietary L‐arginine levels (*p* < 0.05). The highest final body weight (5.06 ± 0.05 g) was recorded in the D‐3 supplemented group, which was significantly higher than those of all other supplemented groups. Specific growth rate followed a similar trend, with the D‐3 supplemented group showing the highest value (3.29%/d), significantly greater than other treatment groups. The FCR improved with increasing L‐arginine levels, and the D‐3 supplemented group had the lowest FCR (1.08 ± 0.02) recorded (*p* < 0.05), followed by a slight increase in higher L‐arginine levels. The protein efficiency ratio was highest in the D‐3 (1.61 ± 0.03) supplemented group, significantly (*p* < 0.05) differing from the other supplemented groups except D‐4, indicating optimal protein utilization at this level. Similarly, BPD peaked (*p* < 0.05) in the D‐3 (23.93 ± 0.50%) supplemented group, compared to other experimental groups. No significant differences were observed in the Fulton condition factor (*K*), HSI, and visceral somatic index across treatments (*p* > 0.05). The condition factor ranged from 1.18% to 1.33%, HSI from 1.54% to 1.77%, and visceral somatic index from 7.16% to 7.46%. Overall, dietary L‐arginine levels significantly influenced growth performance, feed utilization, and protein deposition, with an optimal response observed with D‐3. Higher or lower L‐arginine levels resulted in slightly reduced performance, suggesting an optimal threshold for dietary supplementation. The polynomial quadratic regression analysis based on specific growth rate, FCR, protein efficiency, and BPD rates predicted the optimal requirement of arginine at 3.19%, 3.29%, 3.26%, and 3.33% of diet, respectively (Figures 1, 2, 3, and 4)

**Table 3:** Impact of different dietary levels of arginine on growth performance measurements of coho salmon juveniles.

Parameter	Treatments^1^					
	D‐1	D‐2	D‐3	D‐4	D‐5	D‐6
Survival rate (%)	94.33 ± 1.53	95.67 ± 0.58	97.67 ± 1.15	97.33 ± 1.53	97.67 ± 0.58	97.33 ± 1.53
Initial BW (g)	0.321 ± 0.005	0.318 ± 0.004	0.319 ± 0.006	0.320 ± 0.005	0.322 ± 0.005	0.323 ± 0.004
Final BW (g)	4.51 ± 0.08^c^	4.92 ± 0.04^b^	5.06 ± 0.05^a^	4.93 ± 0.05^b^	4.86 ± 0.04^b^	4.69 ± 0.02^b^
Specific growth rate (%/d)	3.15 ± 0.003^c^	3.26 ± 0.005^b^	3.29 ± 0.01^a^	3.26 ± 0.01^b^	3.23 ± 0.02^b^	3.18 ± 0.01^b^
FCR	1.27 ± 0.04^a^	1.14 ± 0.02^b^	1.08 ± 0.02^c^	1.12 ± 0.01^bc^	1.13 ± 0.01^b^	1.18 ± 0.03^b^
Protein efficiency ratio	1.37 ± 0.04^a^	1.53 ± 0.02^b^	1.61 ± 0.03^c^	1.55 ± 0.01^bc^	1.53 ± 0.01^b^	1.46 ± 0.03^b^
Body protein deposition rate (%)	19.18 ± 0.59^a^	21.77 ± 0.32^b^	23.93 ± 0.50^c^	23.83 ± 0.20^c^	22.87 ± 0.11^ab^	21.58 ± 0.45^b^
K (%)	1.33 ± 0.10	1.22 ± 0.16	1.29 ± 0.15	1.18 ± 0.12	1.25 ± 0.18	1.27 ± 0.14
HSI (%)	1.54 ± 0.05	1.62 ± 0.08	1.77 ± 0.13	1.68 ± 0.07	1.64 ± 0.11	1.60 ± 0.09
VSI (%)	7.33 ± 0.06	7.41 ± 0.11	7.46 ± 0.05	7.22 ± 0.09	7.16 ± 0.05	7.29 ± 0.12

*Note:* Data are presented as mean ± standard deviation, while different superscripts within the same row indicate significant difference between the groups (*p* < 0.05).

Abbreviations: Fulton condition factor; FCR, Feed conversion ratio; HSI, hepatosomatic index; VSI, viscerosomatic index.

^1^Treatments = Dietary L, arginine levels of 2.18% (D‐1), 2.60% (D‐2), 2.97% (D‐3), 3.41% (D‐4), 3.79% (D‐5), and 4.16% (D‐6).

### 3.2. Fish Body Composition

The whole‐body proximate analysis of juveniles showed that dietary arginine levels did not influence (*p* > 0.05) the body fat and ash contents, but protein contents were significantly changed (*p* < 0.05) across the treatments (Table 4). The highest body protein contents were observed in D‐4 and D‐5, followed by D‐3 and D‐6, while the lowest in D‐1 and D‐2. However, the difference of juvenile fish body protein between the D‐1 and D‐2 supplemented groups was not significant (*p* > 0.05). The content increased significantly (*p* < 0.05) with increasing levels of dietary arginine, reaching the highest fish body protein content in the D‐3 supplemented group, followed by a decreasing trend.

**Table 4:** Whole‐body proximate composition on a dry basis of coho salmon juveniles before and after being fed with the different levels of dietary arginine.

Variable	Before feeding trial	After the feeding trial					
		D‐1	D‐2	D‐3	D‐4	D‐5	D‐6
Moisture (%)	77.47 ± 0.28	77.96 ± 0.16	77.88 ± 0.20	77.58 ± 0.12	77.59 ± 0.15	77.26 ± 0.25	77.15 ± 0.19
Crude protein (%)	13.90 ± 0.19^d^	14.01 ± 0.14^c^	14.17 ± 0.16^c^	14.76 ± 0.12^b^	15.23 ± 0.10^a^	14.86 ± 0.11^ab^	14.67 ± 0.09^b^
Ether extract (%)	5.21 ± 0.16	5.26 ± 0.27	5.17 ± 0.23	5.24 ± 0.13	5.16 ± 0.28	5.25 ± 0.19	5.12 ± 0.18
Ash (%)	3.96 ± 0.13	4.01 ± 0.33	3.93 ± 0.24	3.87 ± 0.17	3.96 ± 0.16	4.05 ± 0.23	3.91 ± 0.21

*Note:* Data are presented as mean ± standard deviation, while different superscripts within the same row indicate significant difference between the groups (*p*  < 0.05). Treatments = Dietary L, arginine levels of 2.18% (D‐1), 2.60% (D‐2), 2.97% (D‐3), 3.41% (D‐4), 3.79% (D‐5), and 4.16% (D‐6).

### 3.3. Essential Amino Acid Profile

The whole‐body profile of essential amino acids as % of dry matter of coho salmon juveniles fed with the different levels of arginine is presented in Table 5. The essential amino acid composition of juveniles differed significantly (*p* < 0.05) among dietary groups, particularly for arginine, lysine, and valine. Arginine content increased significantly in the D‐3 (3.37 ± 0.04%), D‐4 (3.39 ± 0.04%), D‐5 (3.36 ± 0.03%), and D‐6 (3.35 ± 0.03%) supplemented groups compared to D‐1 (3.17 ± 0.03%) and D‐2 (3.23 ± 0.03%) supplemented groups (*p* < 0.05). No significant differences were observed among the highest arginine‐supplemented groups (D3–6). The lysine content was significantly higher in the D‐3 (4.42 ± 0.05%) and D‐4 (4.37 ± 0.02%) supplemented groups compared to the D‐1 and D‐2 groups (*p* < 0.05). However, D‐5 and D‐6 supplemented groups showed intermediate values (4.26 ± 0.05% and 4.21 ± 0.04%, respectively) without significant differences from D‐3 and D‐4 groups. The valine content exhibited a significant increasing trend with dietary arginine levels, with the highest concentrations observed in D‐3 (4.69 ± 0.03%) and D‐4 (4.62 ± 0.05%) groups (*p* < 0.05). Similarly, the D‐5 and D‐6 supplemented groups showed slightly lower valine concentrations but remained significantly higher than the D‐1 group (4.25 ± 0.03%). Total essential amino acid content was significantly higher in the D‐3 (27.42 ± 0.07%) and D‐4 (27.15 ± 0.08%) supplemented groups compared to the D‐1 (25.99 ± 0.05%) and D‐2 (26.51 ± 0.06%) groups (*p*  < 0.05). The total essential amino acid content in the D‐5 (26.98 ± 0.08%) and D‐6 (26.66 ± 0.07%) supplemented groups was intermediate and not significantly different from the D‐3 and D‐4 groups, suggesting a potential plateau effect at higher arginine levels. The results indicate that total dietary arginine levels of 2.97%–3.41% were optimal for enhancing essential amino acid deposition, particularly for arginine, lysine, and valine. Further increases in dietary arginine beyond 3.79% did not result in additional improvements, suggesting a saturation effect in amino acid metabolism.

**Table 5:** Whole‐body essential amino acid profile (% of dry matter) of coho salmon juveniles fed with the different levels of arginine.

Essential amino acid profile	Treatments^1^					
	D‐1	D‐2	D‐3	D‐4	D‐5	D‐6
Arginine	3.17 ± 0.03^b^	3.23 ± 0.03^b^	3.37 ± 0.04^a^	3.39 ± 0.04^a^	3.36 ± 0.03^a^	3.35 ± 0.03^a^
Histidine	0.97 ± 0.01	1.02 ± 0.02	1.05 ± 0.02	1.07 ± 0.02	1.10 ± 0.02	0.98 ± 0.03
Isoleucine	2.61 ± 0.02	2.65 ± 0.02	2.71 ± 0.03	2.73 ± 0.03	2.63 ± 0.02	2.58 ± 0.02
Leucine	4.15 ± 0.03	4.17 ± 0.03	4.22 ± 0.03	4.12 ± 0.03	4.26 ± 0.04	4.11 ± 0.04
Lysine	4.12 ± 0.03^b^	4.16 ± 0.03^b^	4.42 ± 0.05^a^	4.37 ± 0.02^a^	4.26 ± 0.05^ab^	4.21 ± 0.04^ab^
Methionine	2.01 ± 0.03	2.06 ± 0.02	2.12 ± 0.04	2.25 ± 0.04	2.16 ± 0.02	2.20 ± 0.03
Phenylalanine	2.44 ± 0.03	2.41 ± 0.03	2.47 ± 0.03	2.38 ± 0.03	2.35 ± 0.04	2.51 ± 0.04
Threonine	2.27 ± 0.04	2.34 ± 0.03	2.37 ± 0.03	2.22 ± 0.04	2.28 ± 0.03	2.31 ± 0.03
Valine	4.25 ± 0.03^c^	4.47 ± 0.03^b^	4.69 ± 0.03^a^	4.62 ± 0.05^a^	4.58 ± 0.05^a^	4.50 ± 0.05^ab^
Total essential amino acids	25.99 ± 0.05^c^	26.51 ± 0.06^b^	27.42 ± 0.07^a^	27.15 ± 0.08^a^	26.98 ± 0.08^ab^	26.66 ± 0.07^b^

*Note:* Data are presented as mean ± standard deviation, while different superscripts within the same row indicate significant difference between the groups (*p* < 0.05). Tryptophan was not analyzed.

^1^Treatments = Dietary L, arginine levels of 2.18% (D‐1), 2.60% (D‐2), 2.97% (D‐3), 3.41% (D‐4), 3.79% (D‐5), and 4.16% (D‐6).

## 4. Discussion

Optimizing dietary amino acid balance is fundamental in aquaculture production due to its direct influence on muscle synthesis, overall growth, and the expression of production potential across fish species [6]. Arginine is an indispensable amino acid in fish, playing vital physiological roles in growth performance and overall health. Therefore, dietary supplementation of arginine supports optimal performance across various fish species [3]. The growth performance measurements include changes in body weights, and survivability is the direct indicator to estimate the impact of dietary interventions in aquaculture commodities [32]. As modern salmonid feeds increasingly rely on high plant protein and low fish meal inclusion, arginine supply from basal ingredients is often inadequate, underscoring the importance of dietary arginine supplementation. In juvenile salmonid, dietary arginine requirements reflect the increased metabolic demands associated with increased protein turnover, osmoregulatory adaptation, and endocrine regulation, indicating the importance of response‐based requirement estimation at this stage. The present study demonstrated that dietary L‐arginine supplementation significantly influenced growth performance and efficiency in coho salmon juveniles, while survival rates remained unaffected. These results indicate that arginine supplementation at the tested levels did not adversely affect fish health or tolerance. Similar findings were reported by Liang et al. [33] and Zhou et al. [34] in juvenile blunt snout bream, where arginine supplementation did not alter survival but significantly enhanced growth performance. Notably, fish fed the D‐3 diet exhibited the highest final body weight (5.06 ± 0.05 g) and specific growth rate (3.29%/d), suggesting that moderate arginine supplementation promotes optimal somatic growth. This result aligns with previous studies indicating the role of arginine as a growth‐promoting amino acid through its involvement in protein synthesis and endocrine regulation [35]. Arginine serves as a precursor for nitric oxide and polyamines, which are known to enhance cell proliferation and nutrient absorption, thereby promoting growth in aquatic species [36]. The FCR followed a similar trend, with the lowest FCR (1.08 ± 0.02) observed in the D‐3 group, signifying enhanced feed utilization efficiency at optimal arginine levels. This suggests that moderate arginine supplementation improves nitrogen metabolism and protein efficiency, which has been previously reported in species such as Nile tilapia and Jian carp [37, 38]. However, the slight increase in FCR at higher supplementation levels (D‐5 and D‐6) may reflect the diminishing returns of excessive arginine intake, potentially due to imbalances in amino acid absorption or metabolic overload [37]. It is also possible that supra‐optimal levels of arginine could lead to competitive inhibition with other cationic amino acids, such as lysine, affecting their absorption and utilization [3]. Dietary arginine requirements of salmonids differ among species, ranging from 1.5% to 2.2% of diet in Salmon salar, 2.2%–2.4% in *Oncorhynchus tshawytscha*, 1.8%–2.5% in *Oncorhynchus kisutch*, and 1.4%–1.9% in *Oncorhynchus mykiss* [16–18, 39, 40]; however, these differences are attributable to species specific variation in growth, protein deposition, and nitrogen metabolism. Physiological functions of arginine are beyond its role in protein synthesis, precursor of nitric acid, polyamines, and creatine, thereby influencing vascular regulation, immune competence, and metabolic pathways. Differences among salmonid species in the activity and regulation of these pathways can contribute to variability in estimated dietary requirements. Species like Chinook salmon exhibit rapid growth and higher anabolic demand indicating higher arginine requirements, whereas Atlantic salmon and rainbow trout exhibit lower requirements consistent with the greater nitrogen use efficiency [16, 17, 39]. Coho salmon indicates an intermediate range of requirement, reflecting its balanced demands for growth and metabolic regulation.

The findings of this study demonstrate that dietary arginine levels significantly influence the proximate analysis and profile of essential amino acids of alevins, particularly in terms of crude protein deposition and amino acid accumulation. Increasing dietary arginine levels improved crude protein content, with the highest values observed in the D‐4 (15.23 ± 0.10%), followed by D‐5 (14.86 ± 0.11%) and D‐3 (14.76 ± 0.12%). These results are consistent with previous research highlighting arginine’s role in enhancing protein synthesis by stimulating the mammalian target of rapamycin (mTOR) signaling pathway, which plays a central role in muscle growth and protein metabolism [41, 42]. The lack of further increases in crude protein beyond D‐4 suggests a metabolic threshold beyond which excess arginine intake may not contribute to additional protein accretion. This aligns with previous reports indicating that excess arginine is catabolized via the urea cycle, leading to increased energy expenditure for nitrogen excretion rather than protein deposition [43]. Crude lipid and ash content remained relatively stable across dietary treatments in this study, suggesting that dietary arginine primarily influences protein metabolism rather than lipid deposition or mineral retention. The crude lipid content ranged between 5.12 ± 0.18% and 5.26 ± 0.27% without significant differences between dietary treatments, implying that excess arginine does not promote lipid accumulation. This is in agreement with studies in other fish species, such as rainbow trout (*Oncorhynchus mykiss*), where arginine supplementation enhanced protein accretion without altering body lipid content [43]. Similarly, ash content remained consistent across dietary groups, indicating that mineral deposition was not significantly affected by dietary arginine levels.

The essential amino acid profile of coho salmon juveniles further supports the role of arginine in protein metabolism. Arginine deposition increased significantly with dietary arginine levels, with the highest values observed in D‐4, 3.39 ± 0.04%. This confirms that dietary arginine directly contributes to tissue arginine accumulation, as observed in previous studies on fish nutrition [44, 45]. Lysine and valine, along with other essential amino acids involved in protein synthesis and muscle development, showed significant increases in response to higher dietary arginine levels. Notably, lysine deposition peaked in D‐3 and D‐4 (4.42 ± 0.05% and 4.37 ± 0.02%, respectively), while valine reached its highest levels in D‐3 to D‐5 (4.69 ± 0.03%, 4.62 ± 0.05%, and 4.58 ± 0.05%, respectively). The positive correlation between arginine, lysine, and valine suggests that dietary arginine enhances overall amino acid utilization and protein turnover. However, the absence of further increases beyond the D‐4 suggests a regulatory mechanism that prevents excessive amino acid accumulation, possibly due to metabolic feedback inhibition or increased catabolism. Previous studies have shown that excess arginine intake can interfere with lysine absorption due to competition for transport pathways, which may explain the stabilization of lysine levels in higher‐arginine diets [46, 47]. The total essential amino acid content exhibited an increasing trend with dietary arginine supplementation, reaching the highest values in groups D‐3 and D‐4 (27.42 ± 0.07% and 27.15 ± 0.08%, respectively). However, further increases in dietary arginine levels in groups D‐5 and D‐6 did not result in proportional enhancements. Comparable trends have been observed in common carp (*Cyprinus carpio*) and channel catfish (*Ictalurus punctatus*), where moderate arginine levels enhanced protein retention, but excessive supplementation led to increased nitrogen excretion [36, 48]. These findings suggest that the physiological response to arginine may follow a similar pattern across fish species, with potential saturation at higher levels. Life stage further modulates the arginine requirements in salmonids, where juveniles normally exhibit relatively higher dietary needs compared with later stages [39]. In coho salmon, the juvenile transition imposes additional metabolic and osmoregulatory demands, which may increase arginine utilization and contribute to variability in reported requirements [17, 18]. Moreover, these observations support the concept that arginine requirements in juvenile salmons are best interpreted using response‐based criteria rather than maximal inclusion levels, reflecting efficient utilization for growth and metabolic functions. From a practical standpoint, formulating aquafeeds with balanced amino acid profiles, especially in terms of arginine–lysine ratios, is crucial for optimizing growth and reducing nitrogen waste. Further research should investigate the interactions between arginine and other amino acids to clarify the metabolic implications of excessive arginine and refine dietary strategies for sustainable aquaculture.

## 5. Conclusion

The present study demonstrates that dietary L‐arginine supplementation significantly influences growth performance, feed efficiency, protein deposition, and amino acid profile in juvenile fish. Among the tested levels, a cumulative dietary arginine concentration of ~3% (D‐3 group) yielded the most favorable outcomes, including the highest final body weight, specific growth rate, protein efficiency ratio, and optimal FCR. Polynomial regression further confirmed the optimal arginine requirement to be in the range of 3.19% to 3.33% based on key growth and utilization parameters. Whole‐body protein content and essential amino acid composition, particularly for arginine, lysine, and valine, were significantly improved at this supplementation level, with no additional benefits observed beyond 3.41%, indicating a metabolic saturation point. These findings suggest that moderate dietary L‐arginine supplementation can optimize nutrient utilization and promote efficient growth in juvenile fish, offering valuable insights for precision feed formulation in aquaculture.

## Author Contributions

**Leyong Yu:** conceptualization, data curation, software, methodology. **Abdur Rahman:** conceptualization, data curation, software, methodology, investigation, project administration, writing – review & editing. **Hairui Yu:** conceptualization, formal analysis, methodology, resources, project administration, supervision. **Govindhrajan Sattanathan:** conceptualization, data curation, methodology, investigation. **Lingyao Li:** conceptualization, data curation, methodology, investigation. **Maida Mushtaq:** conceptualization, software, methodology, writing – original draft. **Muhammad Younus:** investigation, software, supervision, writing – review & editing. **Muhammad Khan:** conceptualization, data curation, software, methodology, writing – original draft. **Muhammad Uzair Akhtar:** formal analysis, software, visualization, writing – original draft, writing – review & editing

## Funding

Funding was provided by the Shandong Provincial Key Research and Development Programs (Major Scientific and Technological Innovation Projects, MSTIP), 2018CXGC0102 and 2019JZZY020710, Scientific and Technologic Development Program of Weifang, 2019ZJ1046.

## Ethics Statement

The ethical approval for the current experiment was granted by the Institutional Animal Care and Use Committee, Institute of Modern Facility Fisheries of Weifang University, China (No. 20220516004).

## Consent

The authors have nothing to report.

## Conflicts of Interest

The authors declare no conflicts of interest.

## Data Availability

Presented data in the manuscript can be obtained from the corresponding author on reasonable request.
